# Developmental Changes in ERP Responses to Spatial Frequencies

**DOI:** 10.1371/journal.pone.0122507

**Published:** 2015-03-23

**Authors:** Carlijn van den Boomen, Lisa M. Jonkman, Petra H. J. M. Jaspers-Vlamings, Janna Cousijn, Chantal Kemner

**Affiliations:** 1 Department of Experimental Psychology, Helmholtz Institute, Heidelberglaan 2, van Unnik building room 16.17, 3584 CS, Utrecht, The Netherlands; 2 Department of Developmental Psychology, Utrecht University, Heidelberglaan 2, van Unnik building room 16.17, 3584 CS, Utrecht, The Netherlands; 3 Department of Cognitive Neuroscience, Section Developmental Cognitive Neuroscience, Faculty of Psychology and Neuroscience, Maastricht University, Universiteitssingel 40, 6200 MD, Maastricht, The Netherlands; 4 Brain Center Rudolf Magnus, Department of Child and Adolescent Psychiatry, University Medical Centre, Heidelberglaan 100, 3584 CX, Utrecht, The Netherlands; Durham University, UNITED KINGDOM

## Abstract

Social interaction starts with perception of other persons. One of the first steps in perception is processing of basic information such as spatial frequencies (SF), which represent details and global information. However, although behavioural perception of SF is well investigated, the developmental trajectory of the temporal characteristics of SF processing is not yet well understood. The speed of processing of this basic visual information is crucial, as it determines the speed and possibly accuracy of subsequent visual and social processes. The current study investigated developmental changes in the temporal characteristics of selective processing of high SF (HSF; details) versus low SF (LSF; global). To this end, brain activity was measured using EEG in 108 children aged 3–15 years, while HSF or LSF grating stimuli were presented. Interest was in the temporal characteristics of brain activity related to LSF and HSF processing, specifically at early (N80) or later (P1 or N2) peaks in brain activity. Analyses revealed that from 7–8 years onwards HSF but not LSF stimuli evoked an N80 peak. In younger children, aged 3–8 years, the visual manipulation mainly affected the visual N2 peak. Selective processing of HSF versus LSF thus occurs at a rather late time-point (N2 peak) in young children. Although behavioural research previously showed that 3–6 year-olds can perceive detailed information, the current results point out that selective processing of HSF versus LSF is still delayed in these children. The delayed processing in younger children could impede the use of LSF and HSF for emotional face processing. Thus, the current study is a starting point for understanding changes in basic visual processing which underlie social development.

## Introduction

The perception of others is a crucial step in the development of social behaviour. Both detailed visual information, such as the face’s wrinkles, and global visual information, such as the proportion between eyes and eyebrows, play an important role in social interaction [1]. For instance, atypical perception of details in Autism Spectrum Disorder is thought to relate to their atypical face perception and social interaction [2–4]. However, there are significant gaps in the knowledge on typical developmental changes in basic perception, that is: outside a social context, of details and global information across childhood. This is particularly the case for development beyond infancy [1,5–7]. Details and global information can be defined in terms of spatial frequencies (SF), where high SF (HSF) carries detailed and low SF (LSF) carries global information. In the current study we investigated the developmental trajectory of selective processing of HSF and LSF in the brain.

**Fig 1:**
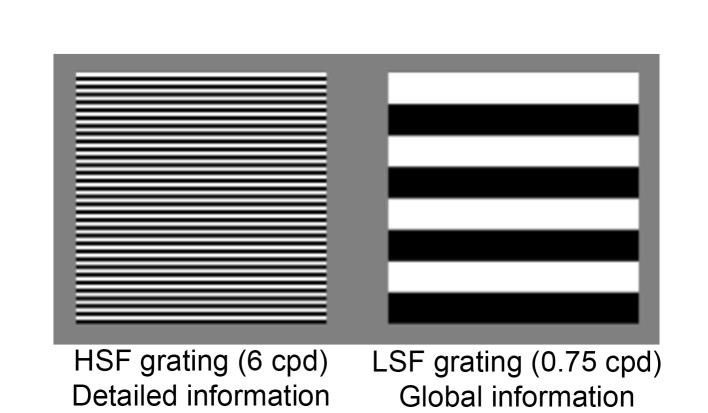
Grating stimuli used in the current study. Detailed and global information are typically defined in spatial frequencies (SF): the grating pattern on the left contains higher SF (HSF) embedding detailed information. The grating on the right contains low SF (LSF), embedding global information.

Previous research mainly used behavioural designs including grating stimuli (see Fig. 1) to study processing of HSF and LSF in children. Newborns discriminate only LSF from noise, but sensitivity to HSF develops rapidly during the first year of life as well (for reviews see [1,6]). Both sensitivity to HSF and LSF show ongoing development after infancy, with milestones between 3 and 12 years of age: sensitivity to HSF has matured at 3–6 years of age, and to LSF at 9–12 years of age [7–10]. Few studies, however, have investigated the neural mechanisms underlying the selective processing of HSF and LSF in the developing brain. In adults, electroencephalography (EEG) studies observed an early peak in brain activity (the N80 peak) around 80 ms after stimulus onset when an image contains HSF, but not when it only contains LSF [11,12]. In contrast, in children aged 3 to 4 years, the EEG signal evoked by HSF images suggest that the N80 peak is not present at all [4]. Instead, a difference in brain activity evoked by HSF versus LSF is only observed later in time in the EEG signal, that is at the P1 peak (~100 ms) and in particular at the visual N2 peak (~200 ms). Thus, the temporal EEG signal characteristics of selective processing of HSF versus LSF may still change significantly across childhood.

Studying the temporal EEG signal characteristics of selective processing of HSF versus LSF provides valuable insight in the moment at which low-level visual brain areas such as V1 process this information and pass it on to higher-level areas, such as the fusiform face area (FFA) involved in face processing. Given the important role of the perception of HSF and LSF in social information processing like reading emotions and identity [13–17], relatively *late* selective processing of HSF versus LSF might restrict processing of social information in higher-level areas.

**Fig 2:**
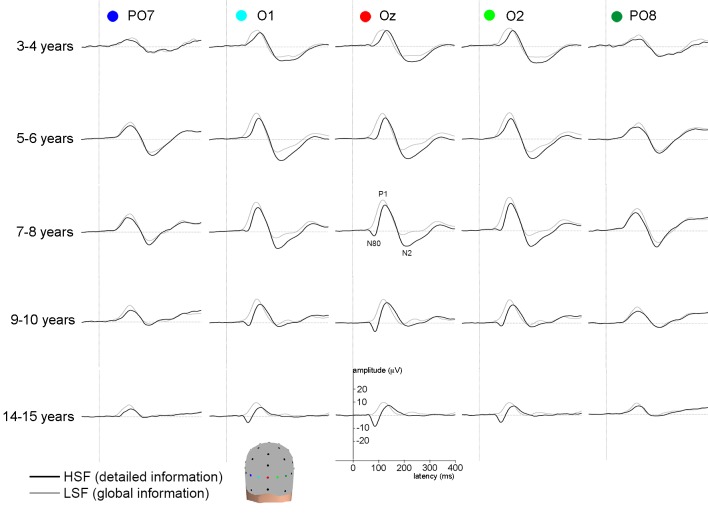
Grand average waveforms for all age groups at occipital electrodes. The grey line represents the grand-averages for LSF (global) stimuli, the black line represents grand averages for HSF (detailed) stimuli. Further analyses were performed on activity measured at the Oz electrode.

In the current study, we investigated developmental changes in the temporal EEG signal characteristics of selective processing of HSF versus LSF in children aged 3 to 10 years, and compared their brain activity to children in which these processes have matured (14 to 15 year-olds) [7,10]. Brain activity evoked by grating stimuli containing HSF or containing LSF (Fig. 1) was measured using EEG, in which evoked Event Related Potential (ERP) peaks were studied. We specifically focussed on early (N80 peak) and later (P1 or visual N2 peaks) visual ERP peaks that have been associated with HSF and LSF processing (Fig. 2) [11,12,15]. For these peaks, we investigated whether peak latency and peak amplitude differed between HSF versus LSF, and whether these differences changed with age. We expected that processing of HSF develops until age 6, and that of LSF until 10 years of age. We suggest that the development of LSF processing would drive developmental changes in *selective* processing of HSF versus LSF until 10 years of age [7–10].

## Methods

### Ethics Statement

The research meets all applicable standards for ethics of experimentation and research integrity. A local ethical committee of the Faculty of Psychology at Maastricht University, The Netherlands, approved the experimental procedure. All parents gave written informed consent for their child’s participation in the study according to the Declaration of Helsinki (2008). Reported data can be accessed via a request to the data handling committee. Please contact the corresponding author for details.

### Participants

The present study included a total of 108 children in 5 age-groups. See Table 1 for number of participants and mean ages per group. Three additional children (3–4 years: N = 2; 14–15 years: N = 1) were excluded from analyses due to poor data quality. The children were recruited from an elementary school in Kerkrade (The Netherlands). To ensure that all children performed in the normal cognitive range (IQ > 85) for their age, children completed an IQ test. All 3–4year-old children successfully completed the Snijders-Oomen Non-verbal intelligence test 2 ½–7 (SON-R) [18], and the other age-groups performed two subtests of the Wechsler Intelligence Scale for Children (WISC-III) [19]: the block design and vocabulary tests. The estimated total IQ score derived from these subtests has a mean reliability of .94 and a mean validity of .91 compared to the complete WISC-III [20]. IQ scores are displayed in Table 1. Additionally, to check for behavioural problems, parents completed the Child Behaviour Checklist (CBCL) [21]. The CBCL is an instrument used for the detection of behavioural problems in children. Three 7–8 year-olds (of 28) and three 9–10 year-olds (of 23) were excluded before experimental testing took place because they scored in the clinical range (> 63) on the Internalizing, Externalizing or Total Problem subscales of the CBCL [21]. Participants had no history of neurological problems and had normal or corrected to normal vision. In the Netherlands, all children get a standard visual check-up at the age of 3 or 4 years.

**Table 1:** Mean Age and Full Scale IQ scores for the five age groups.

	Age in months (SE)	N (# males)	Full Scale IQ (SE)	Range
3–4 years	46.7 (1.6)	21 (7)	121.0 (1.8)	105–138
5–6 years	72.9(1.3)	25 (13)	114.6 (2.0)	90–131
7–8 years	93.4(1.2)	25 (13)	108.9 (2.0)	90–135
9–10 years	119.9(1.9)	20 (10)	102.9 (3.7)	85–147
14–15 years	179.5(1.7)	17 (7)	104.5 (2.3)	85–126

## Stimuli and Task Procedure

Testing took place in a quiet room, where children were positioned in a comfortable chair. All children were videotaped to monitor their behaviour and enable off-line removal of unattended trials. Horizontal (square-wave) black-and-white gratings with either a HSF (6 cycles per degree (c/d)) or a LSF (0.75 c/d) (see Fig. 1) were presented against a grey background. The contrast of all gratings was 100% (Michelson fraction), their luminance was 44 cd/m^2^ and the size was 5.3 degrees of visual angle. Stimuli were presented in random order on a computer with a duration of 500 ms and inter-stimulus interval of 500–1000 ms. A total of 90 stimuli per condition were presented in 3 blocks of 60 trials (30 HSF, 30 LSF). In addition, each block contained 10 different moving and coloured animations (10 per block; duration: 2000 ms), which clearly differed from the grating stimuli. Participants were instructed to maintain fixation and to attend to all pictures. They had to press a response button as soon as they saw an animation figure on the screen and refrain from responding to all other images. This task was used to maintain the participant’s attention during stimulus presentation. After a hit (button press within 3000 ms after stimulus onset) a happy sound (Yoohoohoohoo) was presented, after a miss, a negative sound (boing) was played. Short breaks were given between blocks. At the beginning of the session there was one practice block containing 48 trials and 5 animations.

### EEG recording and pre-processing

ERPs were recorded via an EasyCap containing 39 electrodes. 36 electrodes were placed according to the 10% system (equidistant electrodes): Fp1/2, AFz, Fz, F3/4, F7/8, FC1/2, FC5/6, FT9/10, Cz, C3/4, T7/8, CP1/2, CP5/6, TP9/TP10, Pz, P3/4, P7/8, POz, O1/2, Iz, PO9/10. Three extra electrodes (PO7/PO8, OZ) were added. AFz served as the ground electrode. Additionally, four electrodes placed above and below the left orbit and near the outer canthus of each eye were used to monitor vertical and horizontal eye movements. All electrodes were referenced to the left mastoid online and a second electrode positioned at the right mastoid was measured as an active electrode. Impedances were below 20 kΩ at the start of EEG recording and checked regularly during measurement. EEG was recorded with a 500 Hz sampling rate and a band-pass filter of 0.01–200 Hz.

The EEG data were filtered off-line (0.1–30 Hz). Then data was segmented into epochs of 100 ms pre- to 800 ms post-stimulus. Eyeblinks and horizontal eye-movements were corrected using the algorithm of Gratton et al. [22]. The epochs were then baseline corrected, by subtracting the voltage measured during a prestimulus window (-100 to 0 ms relative to stimulus onset) from the entire waveform, so that the waveform reflects the voltage relative to the average prestimulus voltage. Epochs that contained EEG artifacts were rejected from the data. Artifacts were defined by a voltage change of more than 50 μV per sampling point, a voltage difference of less than 1 μV per 100 ms, or amplitudes below -100 or above 100 μV. Furthermore, trials during which the child did not look at the screen at stimulus onset, as coded using the videotapes, were discarded. All electrodes were re-referenced to an average reference. This reference was the average of activity in the 39 electrodes covering the front, back, left and right side of the head. Separate ERP averages were computed for all subjects, for HSF and LSF gratings. A minimum number of 40 trials per condition were included in the average. Groups differed in the number of trials included (*F*(4,102) = 40; *p*<.001), which was due to a lower number of trials in the 3 to 4 year olds (median: HSF 63; LSF: 64; range: 44–90) compared to the older children (median between 87 and 90; range: 54–90).

**Fig 3:**
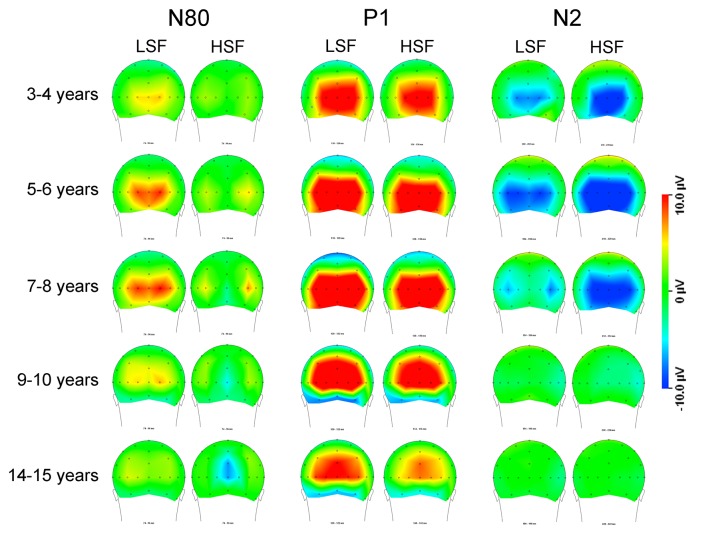
Topographical representations of brain activity, evoked by LSF (global) or HSF (detailed) stimuli at the moment of the N80, P1, and visual N2 peaks.

Based on previous research [11,12,15], we expected activity to be maximal at the Oz electrode during the peaks of interest, which was confirmed for most age-groups and conditions in the current study by visual inspection of ERP peaks and topographies (see Figs. 2 and 3). Therefore, activity at the Oz electrode was chosen for peak analyses. Peaks were automatically detected as the highest point (global maxima) between 50 and 125 ms (N80), 90 and 170 ms (P1) or 150 and 300 ms (visual N2) after stimulus onset and confirmed by visual inspection for each individual average. Peak amplitude and latency were exported for further analyses. Unless otherwise reported, peak amplitude and latency were analyzed using repeated measures ANOVA with SF (LSF; HSF) as within-subject variable and age group (3–4, 5–6, 7–8, 9–10, 14–15-year-olds) as between-subject factor, for each of the peaks separately. An interaction effect of SF and age would indicate that the effect of SF changes with age. In that case, we performed three post-hoc analyses to further study this developmental change. Firstly, to study at which ages a difference in brain activity was observed between SFs, separate t-tests between HSF and LSF stimuli were performed for each age-group. To test whether this difference increased or decreased with age, a difference score was calculated by subtracting peak amplitude evoked by HSF from LSF stimuli, on which univariate ANOVAs were performed using polynomial contrasts to investigate linear or quadratic trends, and repeated contrasts to compare each following age-group (e.g. 3–4 compared to 5–6 year-olds) aiming to determine during which age-ranges specific changes occurred. Finally, to study whether such changes might be due to development of HSF and/or LSF processing, separate univariate ANOVAs for each SF were performed. If applicable, Bonferroni corrections were made for multiple testing. Significance was defined as *p*<.05.

## ERP Analyses and Results

### N80 peak

Visual inspection of peak maxima at age-group average (Fig. 2) and individual level indicated that the amplitude of the N80 peak evoked by LSF and HSF stimuli was close to zero microvolt in 3–6 year-olds, and was for that reason not analyzed in these groups. In older age-groups (7–15 year-olds) the N80 peak was clearly visible as evoked by HSF but not by LSF stimuli, replicating previous studies in adults and older children [11,12,23]. Therefore, we only analyzed the HSF-evoked N80 peak amplitude and latency in 7–15 year old children. In order to still be able to quantify amplitude differences between HSF and LSF at 80 ms in all children, we tested differences in *mean activity* between 75–95 ms for HSF and LSF, similar to Boeschoten and colleagues [12].

#### Amplitude and latency (HSF stimuli in 7–15 year old children)

Two one-way ANOVA’s were performed on HSF amplitude and latency with age (7–8; 9–10; 14–15) as between subject factor. No significant effects of age on amplitude (*F*(2,61) = 1.6; *p* = .216) or latency (*F*(2,61) = 1.1; *p* = .325) were revealed. These results indicate that the N80 peak to HSF stimuli did not further develop after 7 years of age.

#### Mean amplitude activity (HSF and LSF stimuli in all age-groups)

**Fig 4:**
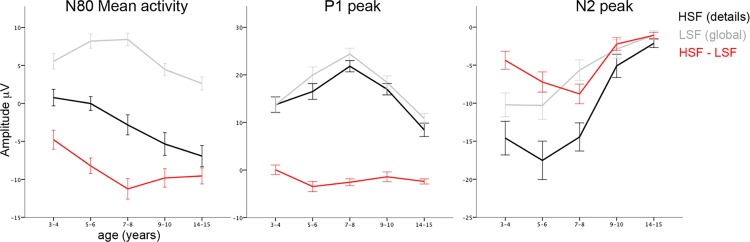
Peak amplitudes measured at the Oz electrode, evoked by HSF (black), LSF (grey) gratings, and the difference-score between HSF and LSF gratings (red) in the investigated age-groups.

Developmental changes were observed for mean amplitude activity, where an interaction between SF and age was revealed (*F*(4,103) = 4.2; *p* = .004). Activity evoked by HSF was more negative than by LSF information in all age-groups (*p*<.01). The difference in brain activity evoked by HSF versus LSF information around the N80 peak increased until 7–8 years of age, confirmed by a quadratic trend (Contrast estimate = 3.2; *p* = .012) showing a significant increase between 3–4 and 5–6 years, and marginal between 5–6 and 7–8 years of age but no changes thereafter (respectively: Contrast estimate = 3.4; *p* = .041; Contrast estimate = 3.0; *p* = .059). Developmental differences were due to changes in processing of both HSF and LSF information at the moment of the N80. As can be seen in Figs. 2 and 4, negative activity increased linearly with age as evoked by HSF stimuli (*F*(4,107) = 6.6; *p*<.001; Contrast Estimate = -6.5; *p*<.001). Evoked by LSF stimuli, positive activity showed a quadratic trend (*F*(4,107) = 7.2; *p*<.001; Contrast Estimate = -3.5; *p*<.001), as it increased in positivity between 3–4 and 5–6 years and decreased between 7–8 and 9–10 years of age (respectively: Contrast Estimate = -2.7; *p* = .036; Contrast Estimate = 4.0; *p* = .002). These results indicate that the different effects of HSF versus LSF information on brain activity as early as the N80 peak increase with age until 7–8 years. From this age onwards, the N80 peak is evoked by HSF but not LSF information.

It should be noted that Fig. 2 suggests that the positive activity evoked by LSF information reflects the rise of brain activity towards the P1 instead of developmental changes in N80 peak activity. As such, the key result of these analyses is the linearly increasing N80 peak with age when evoked by HSF information.

### P1 peak

Visual inspection of peak maxima at age-group average (Fig. 2) and individual level indicated that the amplitude could be reliably detected in all age-groups. Therefore, P1 peak latency and amplitude were analyzed.

#### Latency

LSF gratings evoked faster P1 peak latencies than HSF ones (*F*(1,103) = 137; *p*<.001). No effect of age or interaction between SF and age was revealed (*p*>.1). These results indicate that no development in speed of processing takes place between 3 and 15 years of age.

#### Amplitude

The P1 amplitude differed significantly between SFs (*F*(1,103) = 22.0; *p*<.001), and between age-groups (*F*(4,101) = 12.8; *p*<.001). Furthermore, there was a trend towards an interaction between SF and age (*F*(4,103) = 2.2; *p* = .08). In all but the 3–4 and 9–10 year-olds, the P1 peak was larger as evoked by LSF than HSF stimuli. This difference increased between 3–4 and 5–6 years of age (Contrast estimate = 3.5; *p* = .007), but no specific developmental changes were observed thereafter (*p*>.1). On the contrary, developmental changes were seen for each of the individual SFs: the P1 peak significantly increased for both HSF and LSF until 7–8 years of age, after which it decreased until 14–15 years of age (Contrast estimates for all age-groups and SFs *p*>.05; Figs. 2 and 4). These results indicate that the P1 peak amplitude increased and then decreased with age, irrespective of spatial frequency, and that from 5–6 years onwards brain activity was differently affected by HSF than LSF information at the moment of the P1 peak.

### Visual N2 peak

Visual inspection of peak maxima at grand average per age-group (Fig. 2) and at individual subject level indicated that the amplitude of the visual N2 peak approached zero microvolt in 9–15 year-olds. Here, the lowest peak was detected for analyses purposes, but analyses after 9 years of age were interpreted with caution.

#### Latency

LSF gratings evoked faster visual N2 peaks than HSF ones (*F*(1,103) = 19.0; *p*<.001). No effect of age or interaction between SF and age was revealed (*p*>.1). These results indicate that there was no developmental change in speed of processing.

#### Amplitude

An interaction was observed between age and SF (*F*(4,101) = 7.9; *p*<.001). Overall, in all age-groups HSF evoked larger visual N2 peaks than LSF information (*p*<.01). The difference changed with age in a quadratic trend (Contrast estimate = 4.3; *p*<.001) with a marginal increase between 3–4 and 5–6 years of age (Contrast estimate = 2.9; *p* = .076) and significant decrease between 7–8 and 9–10 years of age (Contrast estimate = -6.6; *p*<.001; all other age-groups: *p*>.1). As is clear from Figs. 2 and 4, this developmental trajectory was due to the decrease in visual N2 peak amplitude that occurred already between 5–6 and 7–8 years when evoked by LSF, but only between 7–8 and 9–10 years when evoked by HSF information (LSF: Contrast estimate = -4.6; *p* = .018; HSF: Contrast estimate = -9.4; *p* = .001). These results indicate that differential effect on brain activity of HSF versus LSF information first increased and then decreased with age, which was due to changes in brain activity evoked by each of the stimuli.

## Discussion

The current study investigated developmental changes in the temporal EEG signal characteristics of selective HSF (embedding detailed information) versus LSF (embedding global information) processing in children aged 3 to 10 years, and compared their brain activity to children aged 14 to 15 years in which these processes have matured. We determined whether peak amplitude and latency of early (N80) and later (P1 and visual N2) ERP peaks differed as evoked by grating stimuli containing HSF versus LSF (Fig. 1). Prominent developmental changes were observed in the early N80 and late N2 peaks (Fig. 2 and 4). More specifically, the difference in N2 peak amplitude for HSF versus LSF was larger in children aged 3 to 8 years compared to older ones. This was due to a decrease in N2 peak amplitude evoked by LSF between 5–6 and 7–8 years of age, followed by a decrease in N2 peak amplitude evoked by HSF between 7–8 and 9–10 years of age. On the contrary, the difference in the early N80 peak amplitude evoked by HSF versus LSF was smaller in children aged 3 to 6 years than older children. This was due to an increasing N80 peak amplitude evoked by HSF until 7–8 years of age. After that age, the N80 peak was only evoked for HSF, not LSF gratings. Of note, fewer trials were included in the average of children aged 3 to 4 years compared to older ones. Although possible influences of fewer trials on the results cannot be excluded, it is unlikely that they fully explain the revealed developmental trajectory. Thus, the current results show that with age, selective processing of HSF versus LSF occurred earlier in time in the EEG signal.

Contrary to our expectations, early processing of HSF becomes evident in the ERPs only at the school-age, and shows a prolonged development. This contrasts previous behavioural results that revealed that processing of HSF has already matured at the age of 3–6 years and of LSF at 9–12 years [6,7]. Our ERP results, combined with previous behavioural results, therefore suggest that even though 3–6 year-old children can already perceive HSF, they show delayed selective processing of HSF versus LSF in the occipital cortex compared to children over 7 years.

Inherently to the employed methodology, the current results do not tell us anything about the neural sources of the current developmental findings for the N80, P1 and N2 peaks. However, based on findings from prior studies in monkeys and human adults, it is possible to speculate about changes in the contribution of neural sources with age [11,12,24,25]. Previous research indicated that the N80 peak might originate from activity in V1 neurons, particularly in parvocellular neurons that respond to HSF. The neural source of the P1 peak is less clear: there are indications that it arises in V1, V2, lateral occipital areas, or a combination of those. Finally, V2 and/or lateral occipital or parietal areas could generate the visual N2 peak. Based on these findings, the increasing N80 peak with age suggests that V1 neurons become more sensitive to HSF with age. It should be noted that V1 neurons already respond to specific ranges of HSF and LSF from birth onwards [26]. However, with age more V1 neurons tune towards a higher sensitivity for more specific ranges of HSF or LSF [26]. The increasing N80 amplitude could indicate that tuning of V1 neurons to HSF continues throughout childhood. The selective processing of LSF versus HSF at the N80 peak and not at the N2 peak in older children could indicate that neurons in V2 or in lateral occipital or parietal areas, associated with the N2 peak, become less sensitive to HSF and/or LSF with age. Thus, with increasing age, both LSF and HSF are processed faster, and therefore possibly with more specificity in lower-level visual brain areas.

The prolonged development of selective processing of HSF versus LSF might affect more complex visual processes that already occur during the N2 peak. This is due to the important role that perception of both HSF and LSF plays in social information processing, such as reading emotions and identity in a face [13,14]. The developmental stage of HSF and LSF processing might constrain face processing: not only processing of spatial frequency and faces, but also the use of specific SFs to derive specific information from a face, differs between children and adults [1,6,7,15,17,27–30]. For instance, whereas adults use LSF to rapidly detect emotional expressions, typically developing children aged 3 to 8 years use HSF [15]. In addition, behavioural recognition of face identity is driven by LSF from 5 years onwards [14], but is optimal for mid SFs after 9 years of age [17]. Moreover, abnormalities in SF processing seem to relate to atypical face processing. For instance, children with disorders such as Autism Spectrum Disorder process SFs differently than controls, and use more HSF information for emotion and identity recognition [4,31,32]. Particularly interesting for future research would be to reveal how the developmental changes in HSF and LSF processing specifically lead to typical and atypical development of face processing and social behaviour.

In conclusion, the current study reveals that selective processing of HSF (detailed information) versus LSF (global information) occurs at a rather late time-point (during the N2 instead of N80 peak) in children younger than 7–8 years of age. The delayed selective processing of HSF versus LSF could impede processing of more complex visual information such as emotional facial expressions. As such, the current study is a starting point for understanding how developmental changes in basic visual processing shape the social development.
